# Arsenic migration and transformation, greenhouse gas dynamics, and their coupled relationships in paddy fields

**DOI:** 10.3389/fmicb.2026.1946222

**Published:** 2026-09-22

**Authors:** Suqing Wu, Chunzhen Fan, Yejian Zhang, Yubi Zhang, Yanan Li

**Affiliations:** 1 School of Transportation Engineering, Huanghe Jiaotong University, Jiaozuo, China; 2 College of Life and Environmental Science, Wenzhou University, Wenzhou, China; 3 Rui’an Hongfu Chemical Fiber Co., Ltd., Wenzhou, China; 4 School of Pharmacy, Guizhou University of Traditional Chinese Medicine, Guiyang, China

**Keywords:** arsenic migration, arsenic transformation, greenhouse gas dynamics, interaction, paddy fields

## Abstract

Paddy fields are a critical nexus where arsenic (As) contamination and greenhouse gas (GHG) emissions pose dual environmental threats. While traditionally studied separately, these processes are biogeochemically interdependent through shared microbial communities, competing electron acceptors, and overlapping responses to agronomic practices. This review provides an integrated synthesis of As and GHG dynamics in rice paddies using a progressive three-tiered framework: from independent mechanisms, to systematic coupling, to integrated management. We first summarize the microbial drivers of As migration and transformation, along with their functional genes and key environmental regulators. We then review the microbial pathways of CO₂, CH₄, and N₂O production and consumption. Centrally, we critically synthesize the coupled interactions between As behaviors and each GHG, identify shared environmental drivers, and evaluate existing co-mitigation strategies and their trade-offs. We conclude by identifying persistent knowledge gaps—particularly the lack of mechanistic clarity on electron partitioning between As(V) reduction and methanogenesis, and the scarcity of simultaneous in-field measurements of As and GHG fluxes—and outline priority research directions for region-specific, co-beneficial management.

## 1 Introduction

Arsenic (As) exists pervasively in the hydrosphere, pedosphere, atmosphere, and biosphere. Owing to its inherent toxicity, As poses a significant threat to human and ecosystem health. Long-term exposure to As not only results in severe toxic effects but also elevates the risk of chronic cancer-related illnesses, including skin cancer, bladder cancer, and lung cancer. Consequently, As is categorized as a Class 1 carcinogen (Hemmat-Jou et al., 2024). Besides, As can severely disrupt plant metabolism, which can lead to death at sufficiently high concentrations of As (Finnegan and Chen, 2012). But many microorganisms have evolved resistance (Uhrynowski et al., 2017).

As originates primarily from natural geogenic, anthropogenic, and biogenic sources. Parent rocks and As-containing minerals—such as carbonates, oxides, silicates, and sulfides—are the main components of natural geogenic sources (Saunders et al., 2018; Valskys et al., 2022). Arsenic biogeochemical transformations are a major exposure pathway of arsenic for living organisms. As shown in Figure 1, volcanoes, fossil fuels, volatilization, and coal smelting can emit As into the atmosphere. Arsenic in the atmosphere can pass into biological systems via inhalation and consumption (Huslina et al., 2024). Anthropogenic activities significantly amplify environmental As contamination. Major anthropogenic sources include As-containing herbicides, wood preservatives, industrial processes, and coal burning. Mining activities in some countries also contribute substantially to As pollution (Sarkar and Paul, 2016). Biogenic sources involve a range of natural processes that release As or modify its speciation in the environment, primarily encompassing microbial methylation and the uptake and accumulation of As by plants (Al-Makishah et al., 2020; Preetha et al., 2023).

**Figure 1:**
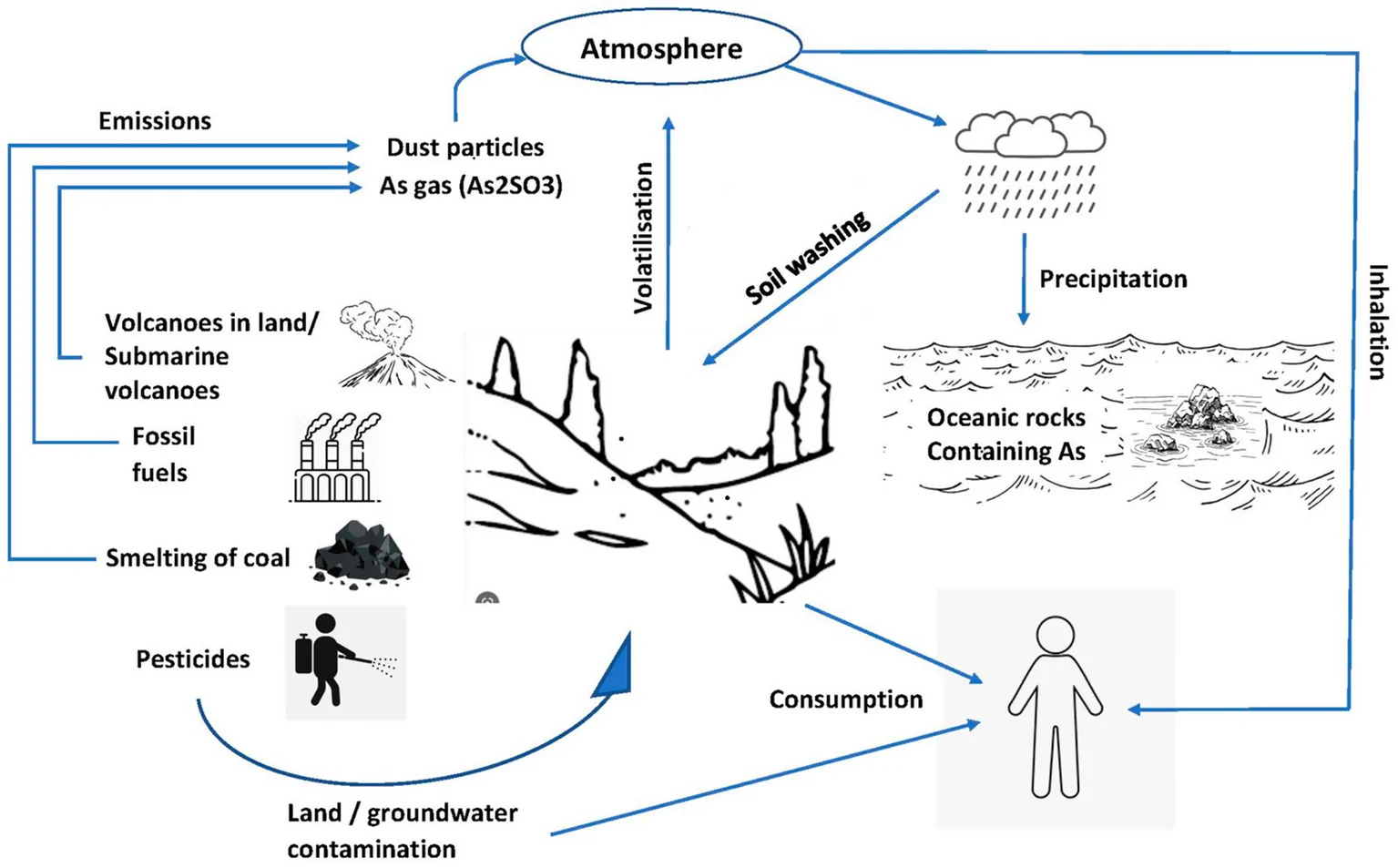
The arsenic cycle. Reproduced with permission from Huslina et al. (2024), Copyright 2024, MDPI.

In nature, As exhibits four primary valence states: +3, +5, 0, and −3, with +5 being the most prevalent. Chemically, As species in natural environments are broadly classified as inorganic or organic forms. Inorganic As undergoes a series of transformations, including reduction, oxidation, biomethylation, demethylation, and conversion into organoarsenical compounds. As(V) and As(III) are two primary inorganic As species, whereas the organic arsenic species that were found most commonly are monomethylarsonic acid (MMA) and dimethylarsinic acid (DMA) (Liao et al., 2022). Different As species exhibit varied toxicity, which could be ordered as MMA(III) > DMA(III) > iAs(III) > iAs(V) > MMA(V) > DMA(V) (Zhang X. et al., 2024).

In the oxygen-free atmosphere before the Great Oxidation Event (GOE), As(III) was the predominant arsenic species in the environment, while As(V) was scarce. The utilization of As(III) as an electron donor by organisms to produce energy was unavoidable (Kulp, 2014). Following the GOE, the majority of As(III) in oceans was converted to As(V). As a newly emerging environmental toxicant, As(V) accidentally enters the cells of most organisms via phosphate uptake systems, pushing early life to develop new strategies to deal with this potentially harmful arsenic species. Given that almost all extant microorganisms rely on ArsB or Acr3 efflux permeases to detoxify As(III), it can be inferred that organisms present before the GOE already had a mechanism to expel As(III). When As(V) emerged as the main form of soluble arsenic, organisms merely needed to convert As(V) to As(III) to develop As(V) resistance. A variety of organisms have independently evolved As(V) reductases (Yan et al., 2017; Zhu et al., 2017). Overall, it is generally believed that all living organisms can detoxify As due to evolution (Ben Fekih et al., 2018; William and Magpantay, 2023). The dominant detoxification route for As is either extrusion from the cell or sequestration in intracellular compartments (Garbinski et al., 2019). Additionally, both inorganic and organic forms of As can be transported out of microbial cells through efflux systems (Zhu et al., 2017).

As biogeochemistry is coupled to many elements, but the interaction between As and the cycling of carbon (C), nitrogen (N), iron (Fe), and sulfur (S) has been most investigated, which influences the biogeochemical cycle of As through multiple pathways. The redox state of iron and pH determine As speciation. Under oxic conditions, As primarily exists as As(V), which is easily adsorbed by iron oxides. Under anoxic conditions, As(III) dominates and is more mobile. Dissimilatory iron-reducing bacteria (DIRB) can release or immobilize As, while nitrate-dependent Fe(II)-oxidizing bacteria can form Fe(III) minerals to coprecipitate As(V). For S, biotic and abiotic redox processes regulate As solubility. Abiotic sulfides form low-solubility arsenic sulfide minerals (e.g., orpiment, realgar). Sulfate-reducing bacteria (SRB) promote As precipitation by forming less soluble As_2_S_3_ or FeAsS, and sulfur-oxidizing bacteria can affect valence transformation of arsenic compounds. For N (mainly nitrate), it supports microbial oxidation of As(III) to As(V) in anaerobic conditions and couples Fe(II) oxidation to facilitate As-Fe coprecipitation, as mentioned before. For natural organic matter (NOM), it can not only adsorb As or form ternary complexes to immobilize As via functional groups (e.g., carboxyl, phenolic hydroxyl), but can also drive reductive dissolution of iron oxides to release As. NOM can also increase the methylation and volatilization of As in paddy soils. Humic acids can directly reduce As(V), and small-molecule organic matter provides electrons for dissimilatory As(V)-reducing prokaryotes (DARPs) to participate in As reduction (Zhu et al., 2017). Critically, the same C, N, Fe, and S cycles that govern As speciation also form the biogeochemical backbone of GHG production and consumption—a convergence that constitutes the mechanistic foundation for their coupling in paddy systems.

Historically, investigations into As transformation have largely focused on microbial reduction, oxidation, and immobilization (Zhu et al., 2017), whereas GHG emission studies have primarily examined environmental controls and mitigation strategies (Qian et al., 2023). Despite sharing the same biogeochemical drivers—C, N, Fe, and S cycles—these two research communities have few cross-communications. However, the mechanistic overlaps outlined above suggest that As and GHG dynamics are not independent but intertwined. For instance, methylotrophic methanogens have been implicated in both CH₄ production and As methylation (Thomas et al., 2011); dissimilatory iron reduction, while mobilizing As from Fe oxides, can competitively suppress methanogenesis by diverting electrons away from methanogenic pathways (Yang et al., 2017); and nitrate fertilization, while enhancing As immobilization via nitrate-dependent As(III) oxidation, simultaneously supplies substrates for denitrification, potentially increasing N₂O emissions. These examples show that single-issue research approaches may produce mitigation recommendations that are internally inconsistent.

Despite these emerging insights, a critical examination of the existing review literature reveals that most syntheses have treated As biogeochemistry and GHG dynamics in separate chapters, with limited systematic analysis of their bidirectional causal links. The specific mechanistic pathways through which As transformations and GHG fluxes influence each other remain largely unexplored in a unified framework. Thus, critical questions still exist: How does As(V) reduction specifically affect CH₄ oxidation kinetics? What are the differential effects of soil organic matter on As mobility versus N₂O production? Which co-mitigation strategies really achieve As(V) reduction and GHG emission decrease, and which inadvertently create trade-offs?

The interdependence of As and GHG dynamics becomes particularly evident when evaluating common agronomic interventions. Alternate wetting-drying (AWD) irrigation, for instance, is promoted for water conservation and CH₄ reduction, yet its effect on As mobility is highly variable—flooding facilitates As(III) release via Fe mineral reduction, whereas drainage may oxidize As(III) to the less mobile As(V), though the net outcome depends on soil properties and drainage duration (Yang et al., 2017). Nitrogen fertilization presents a similar duality: while nitrate can immobilize As through microbial As(III) oxidation, incomplete denitrification can concurrently produce N₂O, introducing a potential trade-off between As stabilization and GHG mitigation (Qian et al., 2023). Beyond these well-recognized examples, organic amendments—though widely used to improve soil fertility—can simultaneously improve methanogenesis and enhance As mobilization by providing electron donors for both Fe-reducing and methanogenic communities, exemplifying another layer of complexity that demands integrated assessment.

For decades, As contamination in rice fields has been a major public concern worldwide. Compared with other primary cereal crops, rice has a higher efficiency in accumulating As, while it also serves as the staple food for populations worldwide (Zhang X. et al., 2024). Paddy fields also act as a major GHG emission source, from which CH_4_ and N_2_O emissions account for 22 and 11% of total agricultural CH_4_ and N_2_O emissions, respectively. The rice rhizosphere stands out as a hotbed of interactions between As and GHG among the key interfaces driving coupled As-GHG dynamics (Qian et al., 2023). Paddy soils are uniquely suitable for studying As-GHG coupling because they experience regular flooding-drainage cycles that alternately favor As(III) mobilization versus As(V) immobilization, and CH₄ production versus oxidation—offering a natural “laboratory” where the two sets of processes are dynamically linked through shared redox switches. Moreover, CH_4_ has received more attention than the other two GHGs. For example, studies have found that low-molecular-weight organic acids (LMWOAs) and other root exudates do not merely solubilize As via competing for adsorption sites on soil colloids and dissolving Fe and Mn minerals (Zhang Y. et al., 2024) but also serve as carbon substrates for methanogens and denitrifiers, directly fueling GHG production. Additionally, iron plaques on root surfaces act as a dual barrier. They sequester As(V) via adsorption (Zandi et al., 2023) while modulating oxygen (O_2_) diffusion into the rhizosphere, thereby influencing the balance between CH_4_ production and oxidation. At the molecular level, recent metatranscriptomic studies have detected co-expression of As-transforming genes (e.g., *arrA*/*arsC2* for As(V) reduction) and GHG-related functional genes (e.g., *pmoA* and *mcrA* for methane oxidation and production) in As-contaminated paddies (Zhao et al., 2024), further reinforcing the notion that microbial metabolic networks link these two processes.

Despite this evidence, the regulatory mechanisms governing As-GHG coupling—particularly electron flow competition between As(V) reduction and methanogenesis, and substrate sharing among functionally distinct microbial communities—remain mechanistically unresolved. Existing studies have predominantly established correlative links between As species and GHG fluxes, but the causal pathways—whether coupling is driven by direct competition for electron acceptors, shared enzymatic machinery (e.g., methyltransferases), or indirect effects through pH/Eh shifts—have yet to be systematically distinguished.

To address these gaps, this review is structured around a three-tiered framework that progressively builds from independent mechanisms to systematic coupling and integrated management. Specifically, we first comprehensively summarize the current state of knowledge on As migration and transformation in rice paddies—covering microbial-driven mechanisms (adsorption, absorption/accumulation, efflux, oxidation, reduction, methylation, demethylation), functional genes, and the key influencing factors, including soil properties, organic matter, minerals, nutrient elements, and periphyton. Second, we review the microbial pathways underlying GHG production and consumption (CO₂, CH₄, N₂O), along with their regulatory environmental factors. Third and centrally, we move beyond parallel summaries to critically analyze the coupled interactions between As behaviors and each of the three GHGs, identify the shared environmental drivers that simultaneously modulate both processes, and evaluate the effectiveness and potential trade-offs of existing co-mitigation approaches. Based on this structured synthesis, we aim to bridge the disciplinary divide between As biogeochemistry and GHG research, highlight critical knowledge gaps, and outline research priorities that can inform region-specific, co-beneficial management strategies for paddy environments.

## 2 Arsenic migration and transformation

This section provides essential background on microbial-driven processes of As migration and transformation, laying the foundation for the subsequent coupling analysis in Section 4. Rather than an exhaustive review, we focus here on mechanistic details that directly intersect with GHG-related microbial pathways—particularly those involving electron transfer, substrate utilization, and shared functional genes.

As in paddy soil originates from As contaminated underground water, the use of pesticides containing As, and mining activities. Rice takes up different As species through rice roots and then translocates them to other parts of the plant. Various forms of inorganic As and organic As are involved in the biogeochemical cycling of As on Earth. As mentioned earlier, they have different toxicity levels, and organisms tend to detoxify As. Many factors can affect As biogeochemical cycling, such as pH, Eh, organic matter, iron, etc. In rice paddy fields, microbial organisms make essential contributions to a variety of As migration (biosorption, bioaccumulation, uptake, and efflux) and transformation (oxidation, reduction, and methylation) mechanisms, as shown in Figure 2. Research has found that the loss of microbial diversity increased the relative abundance of bacteria related to As(V) reduction, leading to mobility of As (Xue et al., 2020; Shi et al., 2021; Jiang et al., 2024). Actually, microbes were found to have a long history of associating with As metabolism and cycling, which dates back to 2.7 million years ago (Sforna et al., 2014).

**Figure 2:**
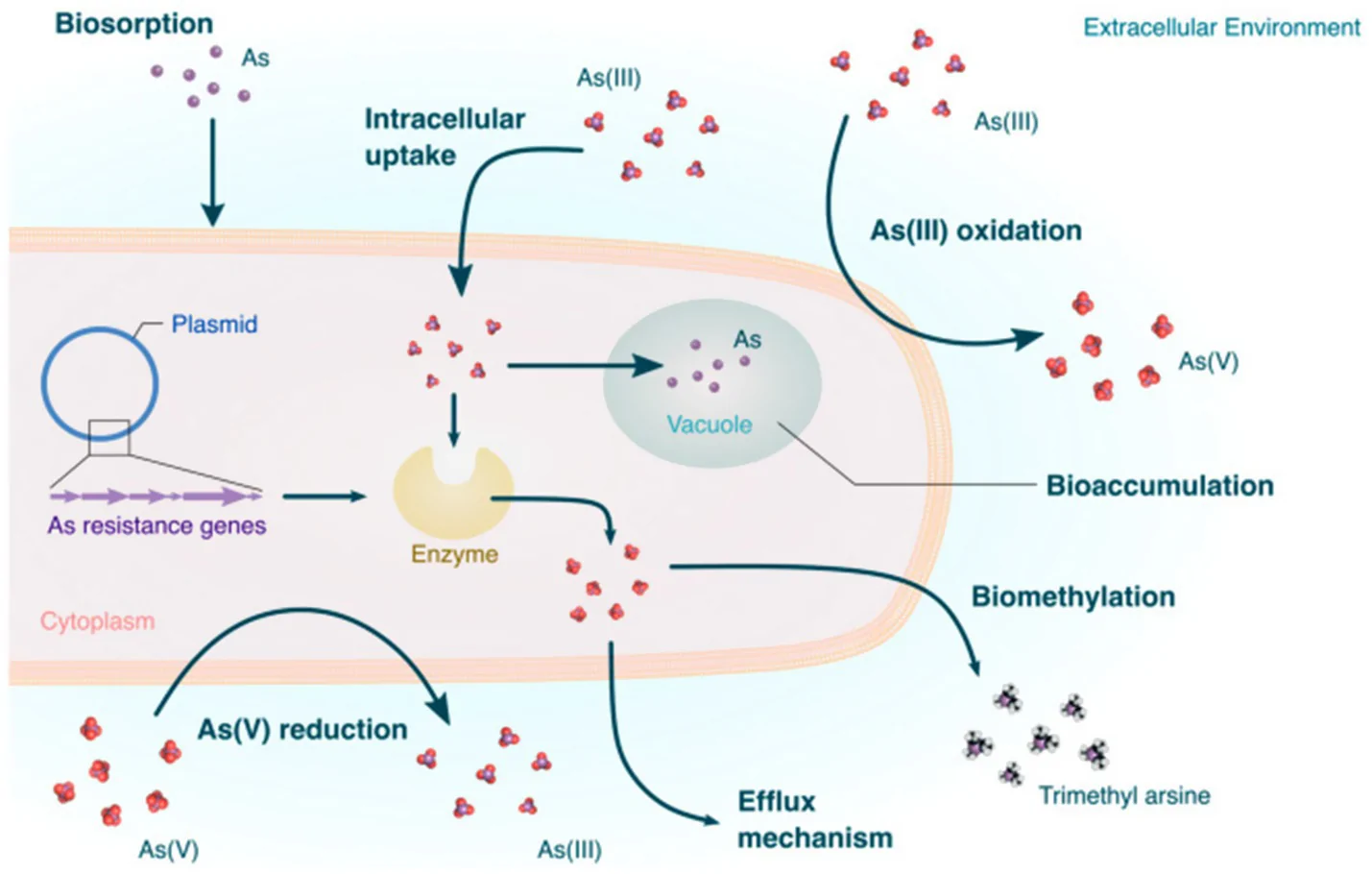
Overview of some underlying mechanisms of microbial tolerance to environmental As. Reproduced with permission from William and Magpantay (2023), Copyright 2023, MDPI.

### 2.1 Microbial-driven arsenic migration

Microorganisms are involved in As migration through adsorption, accumulation, and efflux, but these processes are poorly studied in paddy soils. In paddy soils, adsorption on minerals, accumulation, and efflux in rice are well studied. Understanding the role of microbes in these processes is important for mapping the overall view of As dynamics and benefits As removal; thus, it deserves further investigation.

#### 2.1.1 Arsenic adsorption

Research on As removal from aqueous solutions or the water environment shows that adsorption by biomaterials is primarily driven by the physicochemical interactions between As and functional groups within cell walls. Coordination bonds between arsenic ions and functional groups such as amino, carboxyl, phosphate, and hydroxyl groups on the microbial cell wall enable effective As adsorption. It has been found that *E. coli* can adsorb As(III) from aqueous solution, and the optimum pH for As(III) adsorption is 2.0 (Veglio’ and Beolchini, 1997; Wu et al., 2010; Xing et al., 2024). The adsorption process could be affected by pH, temperature, and As concentration (Wu et al., 2010) and maybe the living status of microbial cells (Huang, 2014). Extracellular polymeric substances (EPS), together with varied organisms like autotrophs and heterotrophs, make up biofilms, which could accumulate heavy metals (Qiu et al., 2024). EPS additionally play dual roles in bacterial As(V) reduction. They function as a reducing agent and simultaneously act as a permeability barrier, controlling access to reduced biomolecules (Zhou et al., 2020). Numerous studies have explored the function of EPS in As accumulation and transformation. For example, compared to *Synechocystis* PCC6803 cells lacking EPS, those with EPS exhibited enhanced As adsorption, As(V) reduction, and As(III) oxidation (Naveed et al., 2020). Moreover, under the stimulation of heavy metals, the secretion of EPS increased (Shen et al., 2018).

#### 2.1.2 Arsenic absorption and accumulation

Arsenic can be taken up and accumulated by microorganisms and plants. Here, we focus solely on bioaccumulating microorganisms. Numerous studies have shown that As enters bacterial cells by mimicking essential nutrients or minerals (Agre et al., 2002; Kang et al., 2012). Aquaporins were reported to help As(III) enter cells of prokaryotes such as *E. coli* (Agre et al., 2002). As for As(V), it can be transported mostly via phosphate (Pi) transporters because of its structural similarity to Pi. Both Pit and Pst phosphate uptake systems were reported to help uptake As(V) in *E. coli* (Kang et al., 2012). Although As can enter the cell, many organisms can still achieve As resistance and detoxification by extruding As from cells or sequestering As in intracellular compartments (Mohsin et al., 2023). As hypertolerant bacterial cells, *Bacillus* sp. strain DJ-1, were isolated from an industrial effluent treatment plant in India. According to the research, they can accumulate both As(III) and As(V) (Joshi et al., 2009).

#### 2.1.3 Arsenic efflux pathways

To extrude As from cells to achieve As resistance and detoxification, microorganisms evolved As efflux pathways. As(III) can be excreted from the cell in many ways, such as the As efflux protein ArsB (Broer, 1993; Lin et al., 2006), protein Acr3p (Bobrowicz et al., 1997), protein Ycf1p (Ghosh et al., 1999), or aquaglyceroporin AqpS (Yang et al., 2005). As for As(V), the common efflux pathway is reduced first to As(III) and excreted through As(III) efflux. However, only one pathway was identified that can extrude As(V) directly from cells. *Pseudomonas aeruginosa* was identified as being able to extrude As(V). This process involved the collaboration of a typical glyceraldehyde-3-phosphate dehydrogenase (GAPDH) encoded by the *gapdh* gene and a permease for organoarsenical efflux (ArsJ). Specifically, GAPDH converts As(V) into 1-arseno-3-phosphoglycerate (1As3PGA)—an unstable organoarsenical. This compound is then transported out of cells via ArsJ and quickly decomposed into As(V) and 3-phosphoglycerate through hydrolysis (Chen et al., 2016). Moreover, the bacterial permease ArsP in *Campylobacter jejuni* is specific for efflux of trivalent organoarsenicals (Chen et al., 2015). ArsK only confers resistance to trivalent arsenicals, including both inorganic and organic forms (Shi et al., 2018).

### 2.2 Microbial-driven arsenic transformation

In the biogeochemical cycling of As, microorganisms are pivotal in As transformation processes, including redox reactions, methylation, demethylation, and volatilization into gaseous arsines (Turpeinen et al., 2002; William and Magpantay, 2023).

#### 2.2.1 As(III) oxidation

Microbe-mediated redox reactions of As contribute significantly to its biogeochemical cycling. As(III) is more mobile and detrimental than As(V) (Hemmat-Jou et al., 2024). *Rhodobacter* strain CZR27, isolated from an As-contaminated paddy soil, was able to oxidize As(III) to As(V) using malate as a carbon source (Wu et al., 2023). As(III) can provide an electron donor for heterotrophic and chemoautotrophic oxidizing bacteria during respiration with oxygen (O_2_) or under nitrate-reducing conditions (Rhine et al., 2005). For example, strain SY, isolated from an As-contaminated paddy soil, could obtain energy by oxidizing As(III) to As(V). This process occurred under both aerobic and anaerobic environments, with O_2_ serving as the electron acceptor in aerobic conditions and nitrate (NO_3_^−^) acting as the electron acceptor in anaerobic conditions (Zhang J. et al., 2015). However, research on water resources shows that the majority of bacteria capable of oxidizing As(III) are heterotrophs. Microorganisms involved in As oxidation include aerobic bacteria and archaea, such as the *Achromobacter*, *Pseudomonas*, *Alcaligenes*, *Thiobacillus*, and *Agrobacterium* genera (Jebelli et al., 2017). The occurrence of As(III) and As(V) depends on pH and redox conditions (Hemmat-Jou et al., 2024). Owing to the challenges posed by rapid shifts in As redox reactions and the absence of a stable As isotopic tracer, the kinetics of microbially mediated As oxidation and reduction, along with the associated rate-limiting factors, remain poorly understood.

#### 2.2.2 As (V) reduction

As (V) reduction is a typical resistance mechanism that helps microbes survive in high As (V) environments. Microbial reduction of As(V) proceeds via two separate pathways: the respiratory pathway driven by *arrA* genes, and the detoxification pathway mediated by *arsC* genes. As(V)-respiring bacteria can reduce As(V) under anaerobic conditions to obtain energy, and *arrA* sequences with a close relationship to *Geobacter uraniireducens* species have been detected in a study of paddy soil. The study also found that *Rhizobiales* and *Pseudomonadales* are involved in detoxification pathways and can facilitate the reduction of As(V) in paddy soils under both aerobic and anaerobic conditions (Zhang S. Y. et al., 2015).

#### 2.2.3 Arsenic methylation

Biomethylation was first observed in fungi and has since been recognized in bacteria, archaea, algae, plants, animals, and humans (Bentley and Chasteen, 2002; Ye et al., 2012). It is considered another way for As detoxification. Microorganisms could use S-adenosylmethionine methyltransferase (ArsM) to catalyze the biomethylation of As(III), and some animals could use arsenic (+3 oxidation state) methyltransferase (AS3MT) to achieve biomethylation. Given that As(III) serves as the substrate for methylation, the prior reduction of As(V) to As(III) is a necessary initial step. ArsM converts inorganic As(III) into methylated species, which eventually end up as pentavalent methylarsenic(V), dimethylarsenic(V), and small amounts of trimethylarsine oxide(V) and volatile trimethylarsine(III). However, the distribution of methylation types is different. For example, both trivalent and pentavalent dimethylarsenic are the most prevalent methylated As species in paddy soil and grains. As methylation prefers anoxic conditions because As(III) is more available. Anaerobes, such as SRB, are considered to enable As(III) methylation (Zhu et al., 2014; Chen C. et al., 2023).

#### 2.2.4 Arsenic demethylation

Unlike methylation, demethylation is also an As detoxification process, while the molecular mechanism remains less well known. An enzyme, ArsI, identified from the bacterium *Bacillus* sp. MD1, can catalyze demethylation of methylarsenite(III) by cleaving the C-As bond (Yoshinaga and Rosen, 2014). ArsI was then found to enable demethylation of methylarsenite(III) under both oxic and anoxic nitrate-reducing conditions in paddy soils (Chen et al., 2021). Demethylation is also possible for dimethylarsenic, yet the specific mechanism governing this process remains unclear. Studies have shown that the H₂-dependent methylotrophic methanogen *Methanomassiliicoccus* is capable of demethylating dimethylarsenic (III) into methylarsenite(III), but it cannot demethylate dimethylarsenic(V) (Chen C. et al., 2023). In addition to As demethylation in rice fields, *Montrachet wine yeast*, *Candida humicola*, and *Mycobacterium neoaurum* are also capable of As demethylation (Crecelius, 1977; Yan et al., 2015). Cyanobacterium *Nostoc* sp. PCC 7120 was also able to demethylate methylarsenite(III) into As(III), which is attributed to the C-As lyase encoded by the gene *NsarsI* (Yan et al., 2015). In both mechanisms, methylarsenate(V) demethylation occurs via a novel two-step pathway: methylarsenate(V) is first reduced to methylarsenite(III), and then demethylation is performed. However, the enzyme responsible for MAs(V) reduction to MAs(III) is still not known (Yoshinaga and Rosen, 2014; Yan et al., 2015).

### 2.3 Genes related to arsenic dynamics

Microorganisms are pivotal to the speciation and mobility of As in soils, sediments, and natural aquatic systems, and these metabolic activities are fundamentally controlled by functional genes mentioned above. The coupling of As biogeochemistry with GHG dynamics further relies on the coordinated expression of As-transforming genes and GHG-related functional genes. Clarifying the correspondence between specific As transformation processes and their associated genes is therefore critical for several reasons. First, it reveals the molecular mechanisms underlying microbial As metabolism, such as the substrate specificity of ArsJ (the sole efflux permease for As(V)) (Garbinski et al., 2019). Second, it provides a basis for interpreting multi-omics data (e.g., metatranscriptomics) to identify key functional taxa linking As transformation and GHG production. Third, it guides the development of targeted remediation strategies, like engineering microbes with functional genes for simultaneous As immobilization and GHG mitigation.

Multi-omics includes metagenomics, metatranscriptomics, metaproteomics, and metabolomics. Metagenomics reveals the taxonomic composition of microbial communities and the abundance of functional genes, laying the foundation for identifying potential functional taxa. Metatranscriptomics, paired with metaproteomics, further captures the temporal dynamics of gene expression and protein synthesis, providing insights into how microbial metabolic activities (e.g., As redox, denitrification) respond to environmental fluctuations. Metabolomics identifies key phenotype- and physiology-associated metabolites (e.g., glutathione, phytochelatins) that serve as biomarkers for linking microbial metabolism to As speciation changes (Zhai et al., 2017).

The application of multi-omics offers a powerful approach to dissect interactions among As-related environmental microbes, addressing a longstanding challenge in which the overwhelming majority of environmental microbes with As functional genes remain unculturable. For example, while shotgun metagenomics identified several As-related functional genes (such as *arsM*, *arsC*, *arrA*, and *aioA*), the PCR-based method only detected the *arsM* gene (Sonthiphand et al., 2024). Notably, multi-omics approaches have unraveled critical coupling relationships between As transformation and biogeochemical cycles of other elements—relationships that would be inaccessible via traditional culture-based methods. For instance, using ROCker models to analyze metagenomic and metatranscriptomic data, the transcriptional activity of *aioA* was found to be significantly correlated with that of denitrification genes (*narG*, *nirK*), and the activity of *arrA*/*arsC2* was correlated with CH_4_ cycling genes (*pmoA*, *mcrA*). This finding directly verifies the coupled relationships between As(III) oxidation and denitrification, as well as between As(V) reduction and CH_4_ oxidation (Zhao et al., 2024). Beyond microbial communities, multi-omics has also revealed that silicon (Si) amendments modulate the expression of genes related to glutathione synthesis, phytochelatin production, and membrane transporters—key pathways that enhance As tolerance in rice and associated microbes (Ahmed et al., 2024). Together, these findings illustrate that multi-omics techniques are indispensable for advancing our understanding of As-microbe interactions in complex environmental systems.

Table 1 summarizes key microbial genes related to major As transformation processes and their special functional characteristics, laying a foundation for subsequent discussions on microbe-mediated As-GHG interactions.

**Table 1:** Genes involved in as transformation and their functional characteristics.

Arsenic transformation process	Involved genes	Special functional notes	Potential links to GHG cycling
As(III) oxidation	*aioA*, *aioB*, *aioX*, *aioS*, *aioR*, *arxA*, *arxB*, *arxB2*, *arxX*, *arxS*, *arxR*, *arxC*, *arxD*, *arsH*, *arsU*, *arsV*, *arsZ*, *moeA*	*arxA* with dual functions: reducing As(V) and oxidizing As(III) *in vitro* (Zhu et al., 2017).	Involvement of *aioA* and *aioB* genes in As(III) oxidation (Li et al., 2020). Significant correlation of transcriptional activity of *aioA* with denitrification genes (*narG*, *nirK*); correlation of *arrA*/*arsC2* was with CH_4_ cycling genes (*pmoA*, *mcrA*) (Zhao et al., 2024).
As(V) reduction	*arrA*, *arrB, arrC*, *arrD*, *arrS*, *arrR*, *ACR1*, *ACR2*, *arsR*, *arsC, arsN*, *arxA*, *GstB*	–	–
As(III) biomethylation	*arsM*	*arsM* as the only gene responsible for As(III) biomethylation in microorganisms (Zhu et al., 2017).	Involvement of ArsM in a methylotrophic methanogen, *Methanosarcina acetivorans* C2A (Wang et al., 2014). Correlation of *Methanosaeta harundinacea* and *Methanosaeta concilii* with a potential *arsM* (Webster et al., 2016).
As(III) demethylation	*arsI*, *NsarsI*	–	–
Arsenic transport (Uptake/Efflux)	*arsB*, *arsA*, *arsD*, *arsJ*, *arsP*, *arsK*, *arsW*, *pgpA*, *aqpS*, *ACR3*, *Ycf1p*	*arsJ* as the sole efflux permease for As(V) (Garbinski et al., 2019). Substrate specificity of *arsP* for trivalent organoarsenicals (Garbinski et al., 2019).	–
Unknown function	*arsO*, *arsT*	Their unknown specific roles in arsenic transformation (Hemmat-Jou et al., 2024).	–

### 2.4 Influencing factors on arsenic behaviors

This subsection outlines key factors that regulate As biogeochemistry in paddy soils, providing essential background for the integrated analysis of shared environmental drivers in Section 5.

#### 2.4.1 Soil properties

As in groundwater could lead to high concentrations of As in paddy soils through irrigation. Besides, As in paddy soils could also increase due to the intensive use of agrochemicals and industrial residues containing As. pH and Eh are important factors controlling As speciation in natural waters. For example, in oxic environments, H_3_AsO_4_^0^ dominates under extremely acidic conditions, while AsO_3_^3−^ may be present in greatly alkaline conditions. Under reducing conditions with pH less than about 9.2, H_3_AsO_3_^0^ will predominate (Smedley and Kinniburgh, 2002). High pH could increase the soil zero-valent S, which benefits the inorganic thioarsenate formation. Thiolation of methylated species is limited mainly by the availability of methylated oxyarsenates, which showed a negative correlation with soil pH (Wang et al., 2020). Moreover, Eh not only determines the existing As species, but it also affects the cycling of Fe, S, N, and dissolved organic carbon (DOC), which is coupled to As cycling in paddy soils (Zhang X. et al., 2024). For example, adsorbed As is released through the reductive dissolution of Iron (Fe) and manganese (Mn) (oxyhydr)oxides, organic matter, and sulfate (Shaheen et al., 2014). In alkaline soils, As(III) is favored for adsorption on surfaces of iron oxyhydroxides and iron oxides, while the sorption of As(V) prefers to happen in acidic conditions. However, this could be significantly affected by soil redox conditions and the solid-to-solution ratio, as well as the type and specific surface area of minerals (Suriyagoda et al., 2018).

Soil pH could also alter the microbial community, resulting in various As behaviors. Increasing pH (>6.5–8.5) showed a decrease in trimethylarsine production by *Penicillium* sp., and trimethylarsine yield reached the highest at pH 6.0, indicating that As methylation and volatilization driven by microorganisms prefer low pH (Huysmans and Frankenberger, 1991). This result was also supported by another study, which found the optimum pH for trimethylarsine production was pH 5 (Cox and Alexander, 1973).

#### 2.4.2 Organic matter

Natural organic matter (NOM) can significantly alter the geochemical processes of As in the rice paddy systems by means of several chemical and microbial approaches. Manure, rice stubble, and roots are the primary sources of organic matter in paddy fields. Organic matter can adsorb As, but it can also enhance As desorption from minerals through competitive adsorption. Furthermore, organic matter can alter the microbial community as a carbon source. For example, biodegradable carbon fuels aerobic respiration, resulting in O_2_-limited conditions and enhancing the microbial reduction of As (Zhang X. et al., 2024).

#### 2.4.3 Soil minerals

Iron (Fe) and manganese (Mn) oxides, redox-active minerals commonly found in rice paddies, strongly influence the biogeochemical cycling of As. Soil-borne iron-containing minerals can adsorb As via coprecipitation. Iron plaque (IP) develops on rice root surfaces and reduces As uptake by rice plants; its formation is influenced by multiple factors including Fe(II) availability, pH levels, and organic carbon content. Moreover, As uptake on IP is also significantly affected by different plant species. It has been found that As uptake on iron oxyhydroxide plaque among 23 rice cultivars is different, which may be due to the considerable variations in IP formation among a range of rice cultivars (Zandi et al., 2023).

#### 2.4.4 Effect of nutrient elements

It has been observed that fertilization applied to paddy soil is not only essential for rice growth but can also exert a specific impact on the migration of As in the soil, which highlights significant potential for controlling As contamination in rice paddies (Zhang X. et al., 2024). Several fertilizers, including nitrogen (N), silicon (Si), selenium (Se), phosphorus (P), sulfur (S), and organic fertilizers, show varied effects on As dynamics in soil. For example, P fertilizer has been shown to compete with As(V) for absorption by rice plants, as both utilize the same transporters in rice roots, and this competition ultimately suppresses As uptake by the plants. Notably, such competition was enhanced at high soil As(V) levels (Jiang et al., 2014).

Nitrate fertilizer can alter the iron redox bacterial community in paddy soils, which in turn oxidizes Fe(II) in soil porewater. This oxidation process triggers the co-precipitation of As with Fe(III) minerals, ultimately lowering As uptake by rice plants (Chen et al., 2008; Wang et al., 2018). Adding ammonium promotes Fe(III) reduction, which in turn releases As(III). This occurs through the collaboration of As(V) reduction and anaerobic ammonium oxidation and ultimately results in As uptake in rice (Chen G. et al., 2023).

S fertilizer can promote the vacuolar sequestration of As in rice, thereby reducing As translocation from roots and leaves to brown rice. It can also hinder As absorption by enhancing iron plaque formation on root surfaces. Furthermore, it can enhance the precipitation of As-S or Fe-As-S compounds using the microbially produced sulfide (Hu et al., 2007; Tuli et al., 2010; Zhang X. et al., 2024).

Si fertilizer has been found to possess the capacity to enhance As desorption from soil particles, achieving this through competitive adsorption, while Si fertilizer can also inhibit As uptake by roots (Lee et al., 2014). This has been proved by other research, which has found that Si fertilizer can successfully reduce the movement of As from soil into plant tissues, mitigate As-induced cellular damage, and enhance rice growth (Ahmed et al., 2024).

Applying Se fertilizer to soil led to an increase in As levels in rice roots, while simultaneously reducing As concentrations in shoots, leaves, and brown rice (Bhadwal and Sharma, 2022). The presence of Se in a system typically helps inhibit the uptake of heavy metals [e.g., As, chromium (Cr)] by iron oxyhydroxide plaque.

#### 2.4.5 Effect of periphyton

Composed of algae, bacteria, and fungi, periphyton spreads widely across wetlands, including paddy fields. Periphyton has been found to increase soil pH and decrease soil Eh, which in turn may lead to elevated levels of total As and iAs(III) in the soil solution (Shi et al., 2017). Many microorganisms in periphyton contain As-metabolism genes and can capture As released from the soil, playing a significant role in As transformation. Moreover, periphyton can accumulate phosphorus (P) in the soil before rice growth while emitting P during late growth, which could indirectly impact As uptake by rice (Zhang X. et al., 2024). However, further studies are needed to better understand periphyton’s roles in As migration and transformation.

The processes described in this section—particularly As(V) reduction (2.2.2), methylation (2.2.3), and demethylation (2.2.4)—involve microbial guilds and electron donors/acceptors that overlap directly with those driving CH₄ and N₂O dynamics. These overlaps will be critically examined in Section 4.

## 3 GHG emissions production and consumption

This section outlines the microbial pathways and regulatory factors governing GHG (CO₂, CH₄, N₂O) production and consumption, providing the complementary background necessary to understand the coupled As-GHG interactions analyzed in Section 4. This section emphasizes processes that share electron donors, substrates, or environmental controls with As transformation.

### 3.1 Microbial-mediated GHG production and consumption

Carbon dioxide (CO_2_) is a key greenhouse gas (GHG) that contributes to global warming, while its impacts can be largely counterbalanced by the net primary productivity of crop plants and their ability to fix atmospheric CO_2_ (Linquist et al., 2012). Figure 3 shows its production process in paddy fields. Moreover, CH_4_ and N_2_O have higher global warming potential than CO_2_. Thus, the majority of existing research about GHG emissions from paddy fields has mainly focused on CH_4_ and N_2_O emissions (Berger et al., 2013). However, all three GHGs should be considered together to develop mitigation approaches for GHG emissions, since CO_2_ emissions vary due to many factors. For instance, CO_2_ emissions from plants are different among varied plant species according to some research (Maucieri et al., 2019).

**Figure 3:**
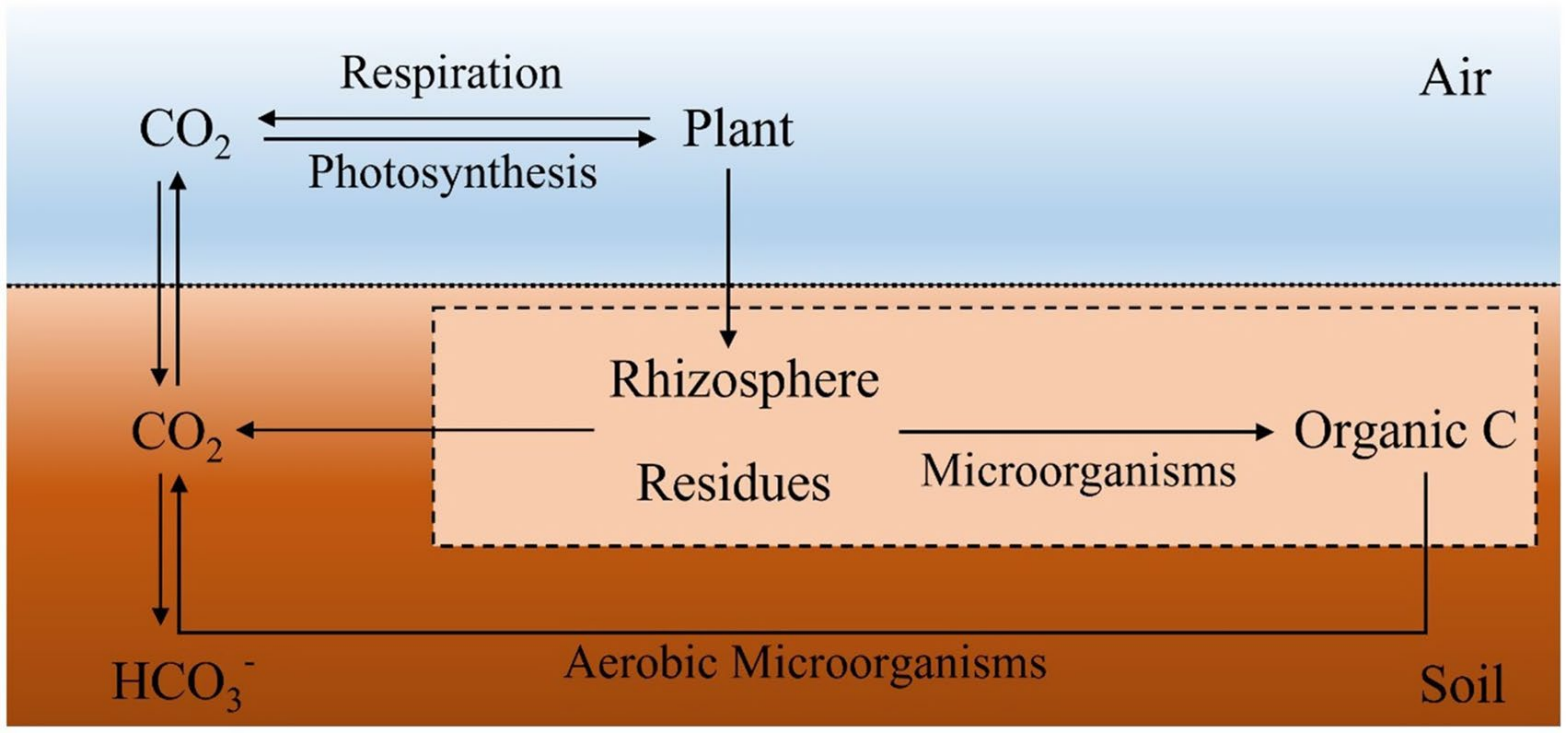
The production process of CO_2_ in rice paddies. Reproduced with permission from Ji et al. (2024), Copyright 2024, Frontiers.

CH_4_ emissions are the net result of three processes—CH_4_ production, oxidation, and transport processes (Qian et al., 2023). As the terminal decomposition process of organic matter, CH_4_ production is mediated by methanogenic archaea that possess the *mcrA* gene, responsible for encoding methyl-coenzyme M reductase (Nwokolo and Enebe, 2025). Methanogens are categorized into three groups by their substrate preferences—hydrogenotrophic, acetoclastic, and methylotrophic—and each group inhabits distinct environments. Hydrogenotrophic methanogens are the most abundant, thriving in deep marine sediments, termite hindguts, and the gastrointestinal tracts of humans and animals. Acetoclastic methanogens dominate anaerobic digesters, rice paddies (Figure 4), and wetlands, while methylotrophic methanogens are prevalent in marine, hypersaline, and sulfate-rich sediments (Lyu et al., 2018). Methane-oxidizing bacteria, commonly referred to as methanotrophs, oxidize methane and diminish its release into the atmosphere via the catalytic activity of particulate methane monooxygenase (pMMO)—an enzyme encoded by the *pmoA* gene (Nwokolo and Enebe, 2025). Methanotrophs are taxonomically divided into two types (Shiau et al., 2018). Low-affinity methanotrophs make up Type I, which requires high environmental CH_4_ concentrations for growth and is mostly located in anaerobic soils (e.g., flooded rice fields) and waterlogged areas. Type II, consisting of high-affinity methanotrophs, can oxidize CH_4_ at low or atmospheric concentrations, with its members primarily inhabiting upland, forest, and well-aerated soils (Nwokolo and Enebe, 2025).

**Figure 4:**
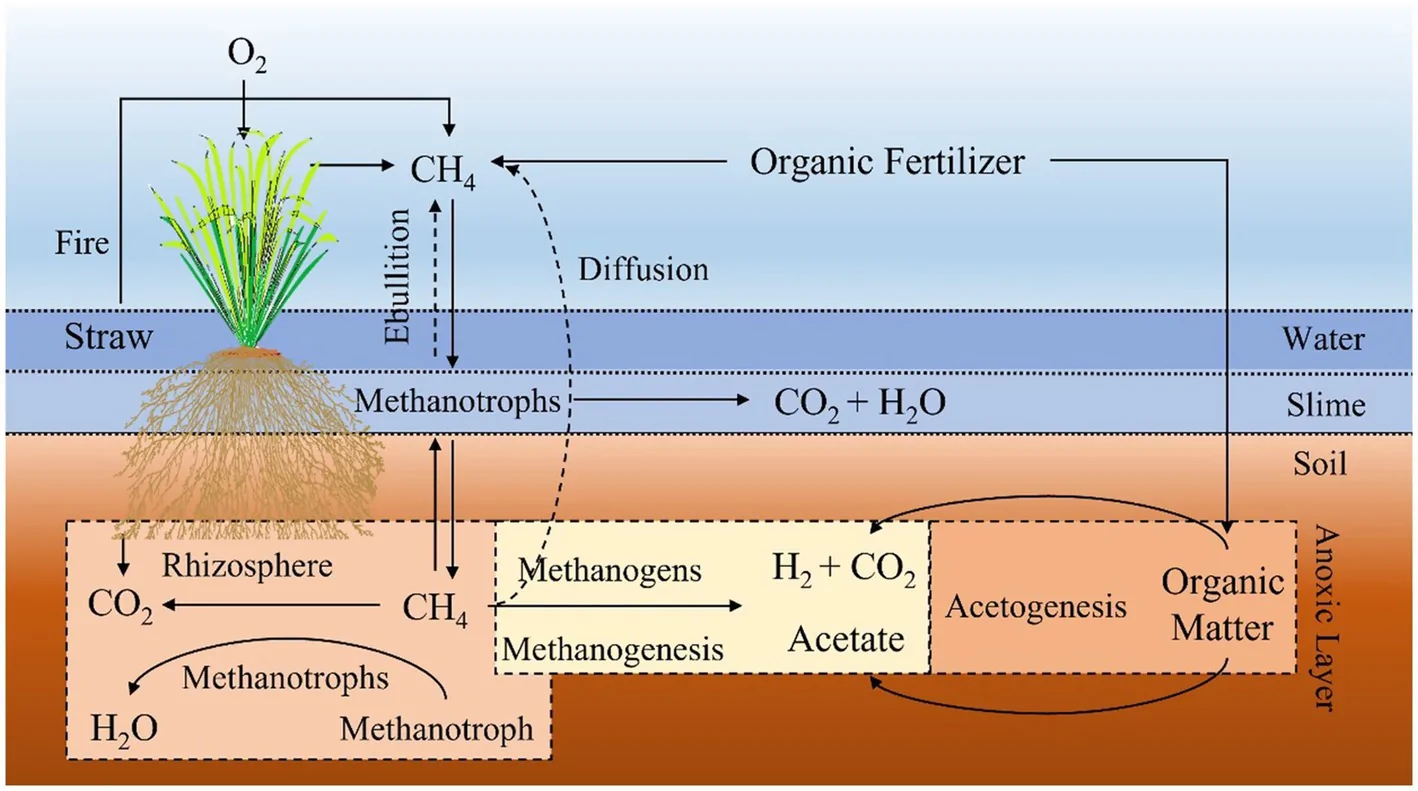
The production process of CH_4_ in rice paddies. Reproduced with permission from Ji et al. (2024), Copyright 2024, Frontiers.

N_2_O emissions from paddy fields mainly result from nitrification of ammonium (NH_4_^+^) under aerobic conditions and from denitrification of nitrate (NO_3_^−^) under anaerobic conditions (Qian et al., 2023) (Figure 5). The conversion of ammonia (NH_3_) to nitrate (NO_3_^−^) via nitrite (NO_2_^−^), known as nitrification, involves three types of microbes: ammonia oxidizers (bacteria, AOB; Thaumarchaeota, AOA), complete ammonia oxidizers (comammox bacteria), and nitrite oxidizers (NOB). Only AOB and AOA are recognized to substantially drive global N_2_O emissions. AOB and AOA can produce ‘hybrid’ N_2_O through the abiotic reactions of metabolic intermediates (NH_2_OH, NO, and NO_2_^−^). Moreover, some AOA species can enzymatically convert NO_2_^−^ to N_2_O under oxic, acidic environments, achieved by expressing a putative cytochrome P450nor. AOB that possess the genetic capacity for nitric oxide reductase (NorB) enzymes can perform nitrifier denitrification, which refers to the enzymatic conversion of NO_2_^−^ to N_2_O via NO. Studies have also found that a type of comammox bacteria can produce N_2_O only through abiotic formation when actively oxidizing ammonia (Stein, 2020). During denitrification, nitrate undergoes sequential conversion to nitrogen gas via the action of four metalloenzymes: nitrate reductases (periplasmic and membrane-bound, encoded by *narG* and *napA*) that reduce NO_3_^−^ to NO_2_^−^; nitrite reductases (cytochrome cd1 and Cu-containing, encoded by *nirS* and *nirK*, respectively) that convert NO_2_^−^ to NO; nitric oxide reductase (*norB*-encoded) that reduces NO to N_2_O; and nitrous oxide reductase (*nosZ*-encoded) that transforms N_2_O to N_2_ (Jenkins and Zehr, 2008). However, roughly one-third of the organisms possess a truncated denitrification pathway, which is deficient in the *nosZ* gene and leads to N_2_O production (Philippot et al., 2011).

**Figure 5:**
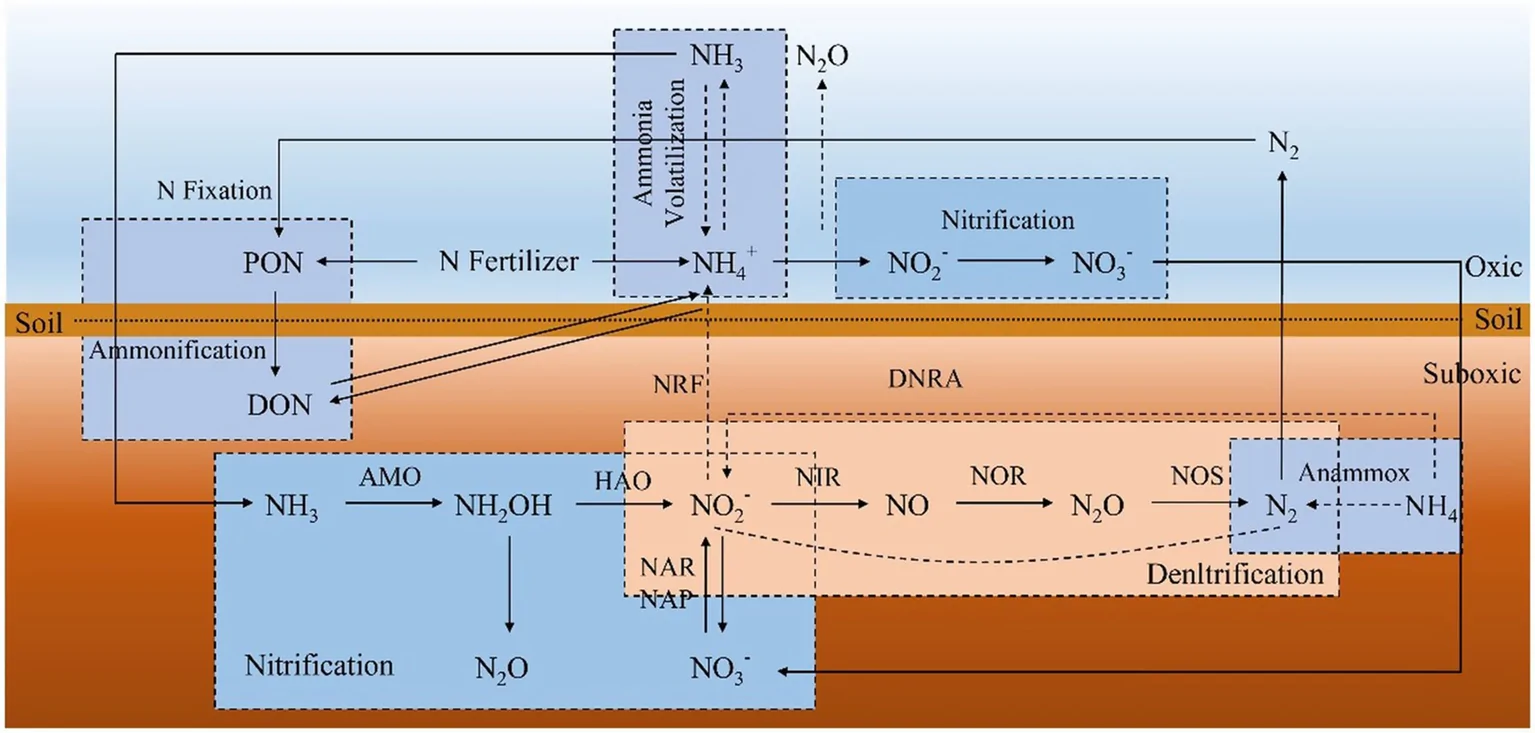
The production process of N_2_O in rice paddies. Reproduced with permission from Ji et al. (2024), Copyright 2024, Frontiers.

### 3.2 Influencing factors on GHG emissions

Various factors can influence GHG emissions from paddy soils, such as soil properties, fertilizer management, water management, rice varieties, tillage practices, and rice establishment methods. This subsection provides a concise overview of these regulators, emphasizing those that also affect As dynamics and are therefore revisited in Section 5.

Soil texture can affect GHG emissions from paddy soils. High clay content of paddy soil may trap CH_4_ into micro-aggregates, resulting in low CH_4_ emissions from soil under flooded conditions (Lagomarsino et al., 2016). However, sandy substrate, which most likely causes strong leaching of NO_3_^−^, may prevent the occurrence of substantial N_2_O production (Berger et al., 2013). Soil pH can also affect GHG emissions. Research has found that a drop in soil pH leads to a progressive weakening of N₂O reductase activity, resulting in an increase of N_2_O (Qu et al., 2014). Besides, the microbial community could also be influenced by pH. For example, in paddy soils, *Methylosinus* tends to dominate CH_4_ oxidation under acidic soil conditions (Shiau et al., 2018), while high pH conditions benefit the activity of anaerobic methane oxidation archaea (ANME) (Hilger et al., 2000). Eh is also an important soil property. CH_4_ emissions from freshwater wetlands decrease as SO_4_^2−^ increases. Three mechanisms were proposed to explain this, and one is that high Eh caused by SO_4_^2−^ suppresses methanogenesis (Linquist et al., 2012).

Different fertilizers affect GHG emissions in varied ways. For instance, compared with the non-fertilized control group, nitrate fertilization resulted in a 1.5-fold increase in total GHG emissions (Grimm et al., 2024). Another study has found that nitrate-based N fertilizers can reduce CH_4_ emissions compared to urea by either preventing redox potential decline or impairing rice growth and root development (Linquist et al., 2012). Notably, even when the total amount of nitrate fertilizer applied was reduced, increasing the frequency of application could still boost overall GHG emissions. Studies have also found that Si fertilizer can decrease CH_4_ and N_2_O production (Das et al., 2021).

Organic matter such as straw and manure supplies substrates for methanogens, which in turn stimulates CH_4_ production. However, CH_4_ emissions respond differently to organic matter type. For instance, the application of livestock manure usually results in a greater increase in CH_4_ emissions compared to straw application. Additionally, long-term addition of organic matter can alter the community composition of methanogens and methanotrophs. This shift leads to a reduction in CH_4_ emissions when straw is used as the organic matter source (Qian et al., 2023). Besides, long-term use of paddy soils influences CH_4_ production. It has been found that older paddy soils produce more CH_4_ than younger ones (León Ninin et al., 2024).

Water management is another critical factor regulating N₂O and CH_4_ emissions. In rice cultivation systems with continuous flooding, N₂O emissions generally remain low across the entire growing period. This is because nitrification and denitrification processes in the soil primarily occur during periods of soil drying and re-flooding, rather than under sustained flooding conditions. Conversely, non-continuous flooding (NCF) practices can effectively boost N₂O emissions by elevating oxygen levels in the soil and stimulating the activity of most nitrogen-transforming microorganisms. However, NCF practices can inhibit CH_4_ emissions in the rice season, typically because they can reduce the abundance and activity of methanogens while enhancing the abundance and activity of methanotrophs (Qian et al., 2023).

GHG emissions also vary among different plant species. Studies have revealed that variety HUW 234 has the highest stomatal frequency in leaves and leaf sheaths, and can emit more seasonal N₂O than Sonalika, HUW 468, and DBW 14. Thus, N₂O emission could be associated with the morphophysiological and anatomical characteristics of plants (Baruah et al., 2012). Plant presence and species did not affect CH_4_ emissions from constructed wetlands, while CO₂ emissions differed notably. Wetlands with *A. donax*, *M. giganteus*, or *P. australis* had higher CO₂ emissions, while those with *C. zizanioides* or no plants had lower CO₂ emissions, confirming plant species’ key role in GHG emissions (Maucieri et al., 2019).

Tillage practices affect soil properties, such as soil structure, moisture, and residue distribution, and each factor can affect GHG emissions. As a core part of conservation agriculture, no-till reduces rice paddy CH_4_ emissions due to lower labile carbon availability, soil redox potential (Eh), and methanogen abundance, yet it exerts no overall influence on either N₂O emissions or rice yield (Qian et al., 2023). Changing tillage from the spring (pre-transplanting stage) to winter (post-harvest stage) during fallow periods promotes the aerobic decomposition of crop residues, reduces net GHG emissions, and increases grain yield in double rice systems (Yang et al., 2018).

Rice establishment methods can significantly affect GHG emissions. For example, compared with conventional-till transplanted rice, both conventional-till direct-seeded and no-till direct-seeded systems result in lower CH_4_ fluxes, though N₂O emissions may increase or remain similar (Chakraborty et al., 2017). Ratooning also affects GHG emissions, with regional variations: in the United States, ratoon crop CH_4_ accounts for ~75% of total growing-season emissions (Linquist et al., 2018); while in China, ratoon seasons have lower CH_4_, reducing GHG emissions compared to double rice systems (Song et al., 2021; Xu et al., 2022). Besides, rotation with different crops affects rice-season CH_4_. For example, rice–rape systems have 61% higher CH_4_ than rice–wheat systems due to more rapeseed residue carbon (Jiang et al., 2022). There are also other factors. For instance, warming also stimulates N_2_O emissions from rice paddies (Qian et al., 2023).

The methanogenic (*mcrA*) and methanotrophic (*pmoA*) pathways described in this section, together with the denitrification (*nirK*/*nirS*, *nosZ*) pathways, are mechanistically linked to As transformation via electron competition for reducing equivalents (e.g., H₂, acetate) and co-occurrence of functional genes in the same microbial taxa. These links are further elaborated in Section 4.

## 4 Coupled and co-occurring relationships between arsenic behaviors and GHG emissions

Paddy soils frequently contain high As levels, which lead to As enrichment in rice grains (Zhang S. et al., 2024), These agroecosystems are also significant sources of GHG emissions (Zhao et al., 2025). The coupling between As transformation and GHG dynamics is fundamentally governed by the thermodynamic hierarchy of electron acceptors that compete for reducing equivalents in anoxic paddy soils. Under anaerobic conditions, the sequential utilization of electron acceptors follows the order of decreasing Gibbs free energy yield: nitrate reduction > Mn(IV) reduction > Fe(III) reduction > arsenate reduction > sulfate reduction > CO₂ reduction (methanogenesis), as demonstrated in paddy soil studies (Wang et al., 2020; Shi et al., 2020; Shi et al., 2022). In flooded rice paddies, Fe(III) and As(V) serve as dominant terminal electron acceptors in the early flooding phase, and their reduction releases Fe^2+^ and As(III) into pore water (Wang et al., 2020) while competing with methanogenesis for available electrons from organic matter (Shi et al., 2020; Shi et al., 2022; Qiao et al., 2018). As Fe(III) pools are depleted and As(V) is reduced to As(III), methanogenesis becomes increasingly favorable (Yuan et al., 2023; Zhang Y. et al., 2024). This thermodynamic hierarchy thus underpins the trade-offs between As mobilization and CH₄ production—electron flow to As(V) reduction suppresses methanogenesis, whereas conditions favoring methanogenesis may also enhance As methylation via shared methyl donors (Thomas et al., 2011). At present, limited research exists on jointly alleviating As-related risks and GHG concerns in paddy fields. Besides, since As are also widely spread in other environments, such as wetlands, marine systems, as well as freshwater and sediment systems, which are also important GHG sources, the relationships between As and GHG dynamics—encompassing direct causal links, indirect effects via shared environmental regulators, and parallel co-occurrence—should be extended to broader environments in future. Figure 6 conceptualizes the coupled biogeochemical interactions between GHG cycling and As dynamics in paddy soil systems, which will be discussed in the following subsections.

**Figure 6:**
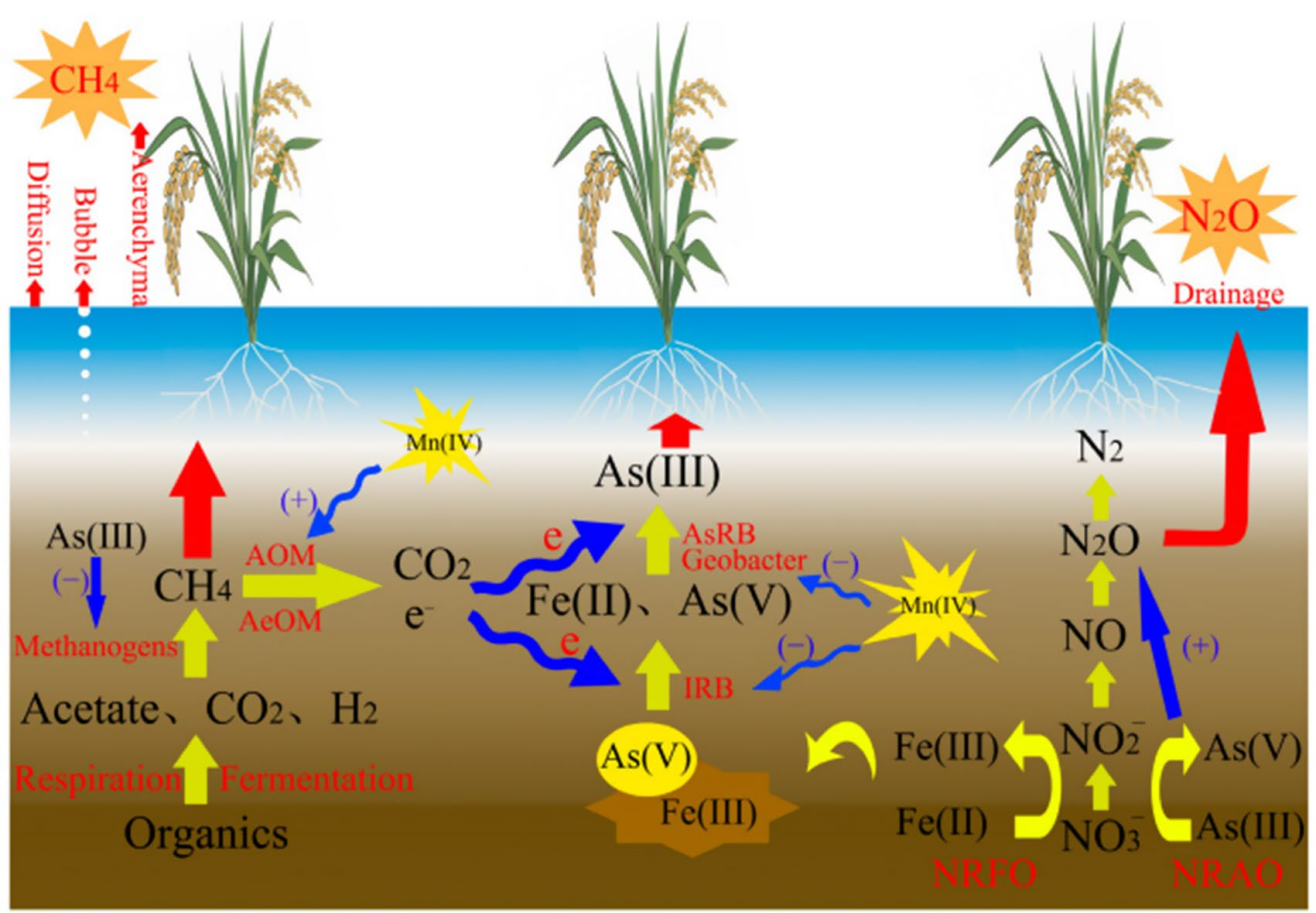
Interaction between GHG production, consumption, and As migration in rice paddy fields (AOM: Anaerobic methane oxidation; AeOM: Aerobic methane oxidation; NRFO: Nitrate reduction coupled with Fe(II) oxidation; NRAO: Nitrate-reduction coupled with As(III) oxidation; AsRB: As (V)-reducing bacteria; IRB: Iron-reducing bacteria). Reproduced with permission from Qi et al. (2025), Copyright 2025, MDPI.

### 4.1 Interactions between arsenic behaviors and CO_2_ emission

According to a pot experiment, As stress significantly decreased the photosynthesis rate, stomatal conductance, and internal CO_2_ in wheat (Ali and Perveen, 2020), indicating CO_2_ emissions from plants would be inhibited by As stress through direct phytotoxic effects on photosynthetic machinery.

A microcosm experiment confirmed that heavy metal (including As, Pb, Cu, Zn, and Cr) pollution can inhibit CO_2_ emission initially due to impeded organic matter decomposition, though this inhibition was not evident in field examinations (Enya et al., 2020). Conversely, a study in mangrove wetlands proposed that total carbon accumulation in soil exerts a strong regulatory effect on heavy metal toxicity, ultimately reducing their toxic impacts and leading to higher CO_2_ emission (Ma et al., 2021). These contrasting observations suggest that the apparent As–CO₂ relationship is largely mediated indirectly through soil carbon dynamics and physicochemical properties, rather than reflecting a direct causal link between As and CO₂ fluxes. The research also showed the opposite dynamic of CO_2_ and CH_4_ fluxes due to heavy metal contamination (Ma et al., 2021), thus, both two GHGs should be taken into consideration to evaluate the influence of heavy metal contamination on GHG fluxes because CH_4_ has a higher global warming potential than CO_2_. Moreover, researchers proposed that pH, Eh, or EC are also significant factors in the carbon dynamics of soil compared to heavy metals, indicating that these factors could also affect the interaction between As and CO_2_ emissions, which requires further investigation (Enya et al., 2020).

### 4.2 Interactions between arsenic behaviors and CH_4_ emission

Methylation of As is highly involved in CH_4_ production. During methylotrophic methanogenesis, methylcobalamin [CH₃Cob(III)] provides methyl donors for As methylation (Thomas et al., 2011). Moreover, ArsM has been identified in a methylotrophic methanogen, *Methanosarcina acetivorans* C2A (Wang et al., 2014). Two acetoclastic methanogens, *Methanosaeta harundinacea* and *Methanosaeta concilii,* were found to possess a potential *arsM*. Therefore, methanogens are closely linked to As methylation and CH_4_ emissions. High amounts of As volatilization are expected to occur under anaerobic conditions with high CH_4_ production in a methanogenic mesocosm set up with cow dung and As waste from a drinking water treatment system (Webster et al., 2016). However, a contradictory result was found that methanogenesis inhibits As biovolatilization in flooded paddy soils, attributed to the broader range of terminal electron acceptors [e.g., Fe(III), SO_4_^2−^] leading to a greater diversity of As methylation metabolisms. Additionally, they found that methanogens are associated with just a small fraction of As biomethylation and volatilization processes (Zhang and Reid, 2022).

As demethylation also involves active methanogens. Studies have found that the collaboration of *Clostridium* and *Methanomassiliicoccus* can alleviate straighthead disease by decreasing DMA accumulation in rice plants. *Clostridium* is responsible for the reduction of dimethylarsenic(V) to dimethylarsenic(III), while *Methanomassiliicoccus* demethylates dimethylarsenic(III) and provides H_2_ for the growth of *Methanomassiliicoccus* (Chen C. et al., 2023).

CH_4_ is known to drive microbial reduction of diverse oxidants in both aerobic and anaerobic conditions (Devol and Ahmed, 1981; Kits et al., 2015; Zheng et al., 2020). Anaerobic oxidation of methane (AOM) thus has the potential to be coupled to As(V) reduction (Figure 6). It has been shown that AOM coupling with As (V) reduction was responsible for 26 to 49% of total As release from soils in microcosm incubations using natural wetland soils (Shi et al., 2020).

Moreover, aerobic methane oxidation (AeOM) carried out by aerobic methanotrophs could also couple to As(V) reduction carried out by *Burkholderiaceae*. In this process, formate-linked AeOM couples to the reduction of As (V), acting as the interspecies electron carrier. These coupled bioprocesses also occur in real-world environments, contributing up to 76.2% of soil-As mobilization into pore waters in oxygen-containing topsoil (Shi et al., 2022).

### 4.3 Interactions between arsenic behaviors and N_2_O emission

The relationships between N₂O emission and As cycling—including both direct process coupling (e.g., NRAO) and indirect co-regulation via shared microbial genes— have received increasing attention. While N_2_O is emitted from various niches, research on its interaction with As has mainly focused on agriculture, especially paddy soil. Rice paddies are considered a main agricultural source of GHG and account for up to 48% of GHG emissions from cultivated lands. Rice production requires large amounts of N fertilizer, which is the most important N_2_O emission driver (Qian et al., 2023; Qi et al., 2025).

Research has found that immobilization of 75–82% aqueous As(III) is achieved through the coupling of nitrate reduction and As(III) oxidation (NRAO), though this process boosts N₂O emissions by 2.3 times owing to incomplete denitrification (Chen et al., 2025). As shown in Figure 6, during NRAO, nitrate serves as an electron acceptor that directly oxidizes toxic As(III) into less mobile As(V). In the NRFO pathway, nitrate oxidizes soluble Fe(II) into Fe(III)-(hydr)oxides, which sequester arsenic via adsorption and co-precipitation. Nevertheless, incomplete nitrate denitrification may trigger unintended N_2_O emissions, creating a mitigation trade-off. Beyond nitrate, manganese-bearing minerals function as high-redox-potential oxidants to limit As release by oxidizing Fe(II) and As(III), and they can act as electron acceptors or photocatalysts to reduce methane production in paddies (Qi et al., 2025). However, in neutral As-contaminated paddy soil, the combination of As(III) oxidation and DNRA not only cuts down N₂O emissions but also boosts the reduction of NO_3_^−^ to NH_4_^+^ (Gan et al., 2024). Therefore, further research should explore how different N₂O production pathways causally link to As cycling in various environments with different types of N and As.

N_2_O is an intermediate that can be reduced to N_2_ by nitrous oxide reductase (NosZ), encoded by *nosZ,* existing in denitrifying bacteria but not in denitrifying fungi (Castellano-Hinojosa et al., 2021). Several chemoautotrophic bacteria in paddy soils are capable of oxidizing As(III)—*Azoarcus* sp. DAO1, *Sinorhizobium* sp. DAO10, and *Paracoccus* sp. SY.—contain the genenos for nitrous oxide reductase. *Azoarcus* sp. has been shown to possess not only the *aioA* and *aioB* genes involved in As(III) oxidation but also a complete suite of denitrification-related genes (mainly *napA*, *nirK*, and *nosZ*). It has been found that *aioA* and *nosZ* genes increased after As(III) addition, consistent with the lower N_2_O emissions in As(III) treatment (Li et al., 2020). This represents a process-level coupling at the genetic level, where As(III) oxidation and N₂O reduction are functionally linked within the same microbial taxa through co-expression of their respective functional genes, rather than a coincidental co-occurrence.

## 5 Shared environmental drivers and their simultaneous, sometimes opposing, effects on arsenic dynamics and GHG emissions

Having outlined the respective influencing factors for As (Section 2.4) and GHG (Section 3.2), this section moves beyond parallel summaries to explicitly analyze how each shared driver simultaneously—and often in opposing directions—modulates As speciation and multiple GHG fluxes. Organic matter not only promotes the biological respiration of As(V) but also serves as a substrate for fermenters. These fermenters then produce the precursor required for CH_4_ production (Qiao et al., 2018, 2019). Therefore, a carbon source is important for both processes. Soil pH is a critical factor for As(V)-dependent CH_4_ oxidation. As(V)-dependent CH_4_ oxidation was inhibited under acidic conditions in a short-term experiment, while the inhibition effect was not obvious when the acidic conditions were prolonged (Yuan et al., 2023). Moreover, rainwater-borne H_2_O_2_ can react with Fe(II), triggering a Fenton-like reaction that inhibits As(V)-reducing microbes but promotes As(III)-oxidizing microbes, leading to a net increase of As(V). In turn, this reduces the uptake of soil As by rice roots, lowering As accumulation in rice grains. And rainwater-borne H_2_O_2_ can also lead to a reduction in CH_4_ and N_2_O by influencing microbes in soil (Qin et al., 2023). As for soil texture, it influences As migration primarily through the distribution of Fe/Mn/Al oxides that bind As (Sadiq, 1997), while also modulating GHG emissions by affecting CH₄ entrapment in micro-aggregates and NO₃^−^ leaching (Khan et al., 2024; Qi et al., 2025)—exemplifying how a single physical property exerts divergent effects on the two environmental issues.

## 6 Co-mitigation approaches for arsenic mobility and GHG emissions

Current mitigation research shows an obvious imbalance: a wealth of studies focuses on arsenic remediation, while GHG reduction measures are relatively understudied. Those strategies that can lower GHG emissions and As pollution at the same time are rarely reported.

To move beyond a simple inventory of available techniques, we explicitly link each co-mitigation strategy discussed below to the mechanistic couplings identified in Sections 4–5—specifically (i) direct process-level couplings (e.g., electron competition between As(V) reduction and methanogenesis), (ii) indirect effects via shared environmental drivers (e.g., redox modulation by water management), (iii) co-occurrence patterns (e.g., parallel stimulation of As methylation and methanogenesis by labile carbon inputs). This mechanistic grounding allows a critical evaluation of why a given strategy may succeed, fail, or produce unintended trade-offs.

Specific agronomic practices can influence As behaviors in paddy fields, thereby effectively reducing As pollution in rice (summarized in Figure 7). For example, breeding high-yield rice cultivars with low root As uptake and low GHG emission potential offers a long-term genetic solution. Optimized water management, including region-adapted alternate wetting-drying regimes and precise water-level regulation, can strengthen soil oxidative conditions while avoiding water-stress damage during critical rice growth stages (Qi et al., 2025). These water management strategies operate primarily by modulating soil redox potential—a shared environmental driver (Section 5)—thereby simultaneously suppressing methanogenesis (by limiting anaerobiosis) and As(III) mobilization (by promoting As(III) oxidation to less mobile As(V)). Continuous flooding leads to higher dissolved As concentrations in soil solution than continuous aerobic conditions, which not only facilitates the release of Fe and As in soil pore water, but also increases As(III) and methylated As species in rice grains relative to aerobic treatments (Wang et al., 2019). Intermittent irrigation enhances soil aeration and modulates soil redox potential, thereby reducing As migration and accumulation in rice grains (Zhang X. et al., 2024). Notably, these redox-regulation strategies provide dual ecological benefits. A study has further demonstrated that moderate alternate wetting-drying practices can improve rice quality by reducing As accumulation in grains while simultaneously lowering CH_4_ emissions from paddy fields (Yang et al., 2017). Here, the dual benefit arises from the shared redox control: drainage interrupts both Fe(III)- and As(V)-reducing conditions that fuel CH₄ production, while also shifting As speciation toward the less mobile pentavalent form (Figure 8).

**Figure 7:**
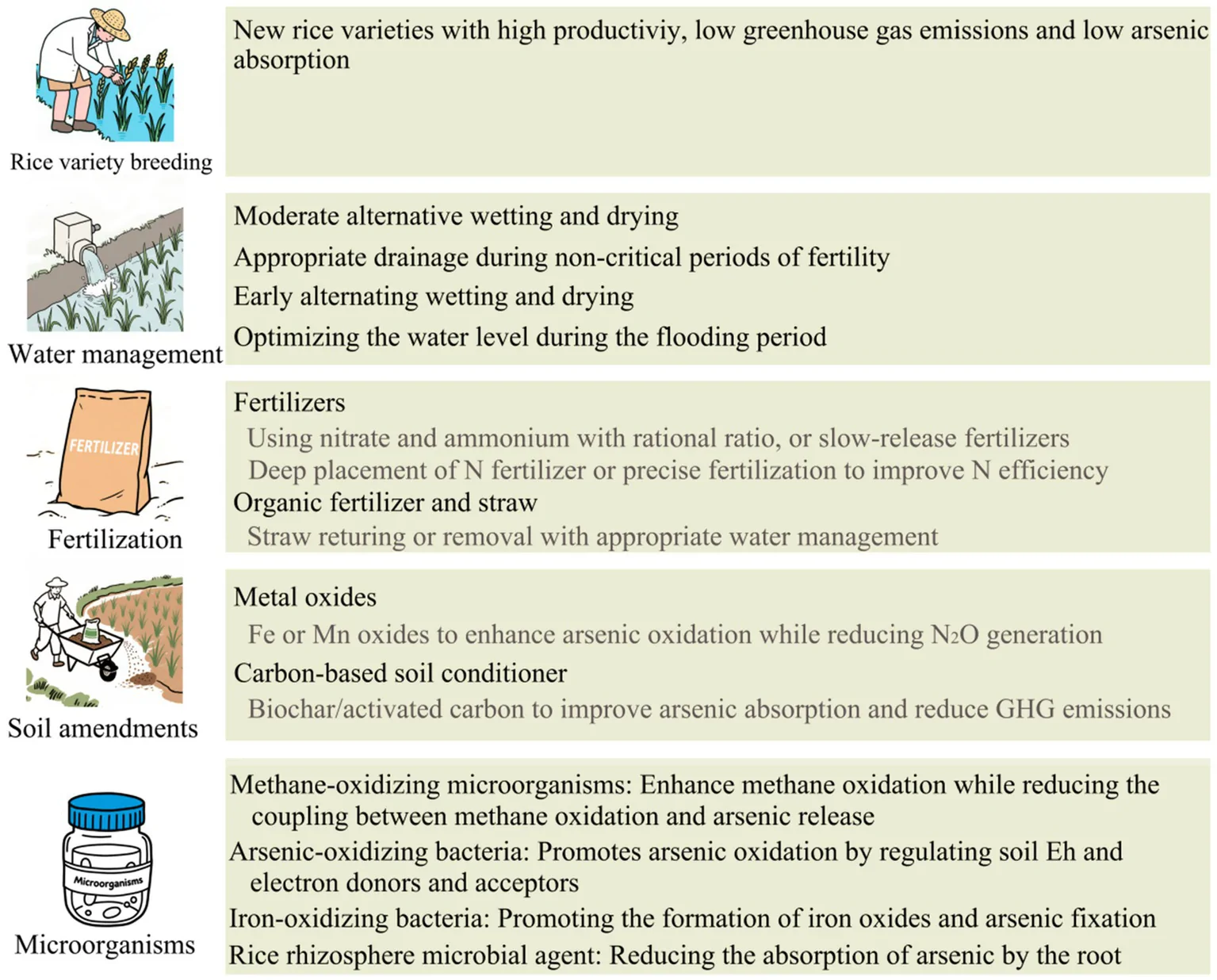
Potential measures for simultaneously mitigating GHG emissions and As migration in rice paddy fields. Reproduced with permission from Qi et al. (2025), Copyright 2025, MDPI.

**Figure 8:**
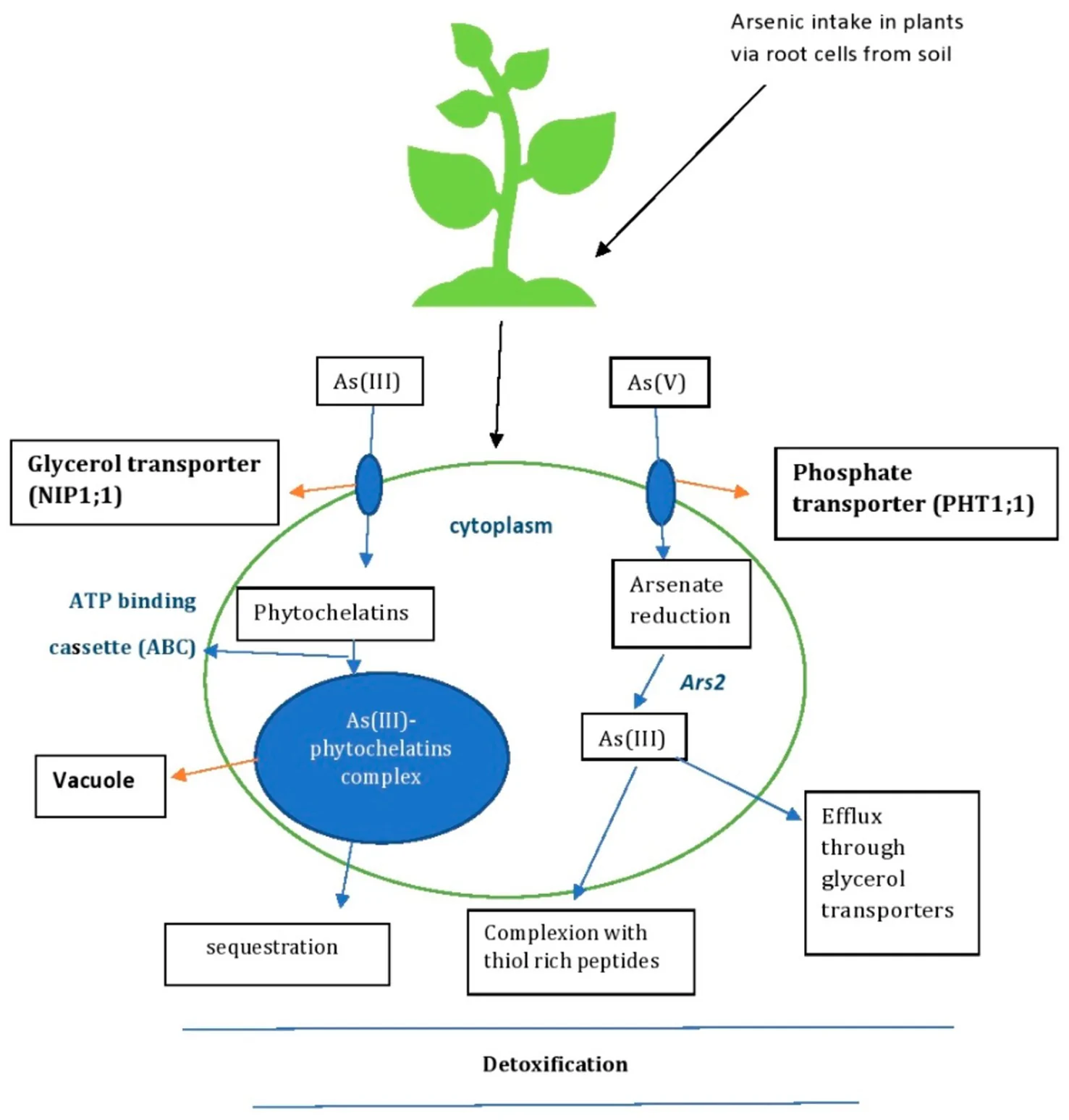
Mechanisms of As detoxification by plants—phytoremediation. Reproduced with permission from Preetha et al. (2023), Copyright 2023, MDPI.

Microbial-oriented remediation, including the application of methane-oxidizing, arsenic-oxidizing, and iron-oxidizing functional bacteria as well as rhizosphere microbial agents, offers a promising eco-friendly approach to concurrently limit As mobility and GHG emissions in paddy ecosystems (Qi et al., 2025). Both natural and genetically modified organisms (GMOs) can be harnessed for remediation purposes. For example, a study has shown that employing soil microbial fuel cells (sMFCs) can reduce Fe and As release into paddy soil porewater (Gustave et al., 2018). This approach mechanistically intervenes in electron flow: sMFCs divert electrons away from dissimilatory Fe(III) and As(V) reduction, thereby competing with methanogenic electron sinks and simultaneously lowering both As mobility and CH₄ production. Certain viruses that infect methanotrophs have large genomes encoding *pmoC*—a gene that may enhance methane oxidation by stimulating the activity of the infected methanotrophs. A study found that while extracellular free viruses promoted methane-mediated As mobilization, viruses induced by mitomycin C exert an inhibitory impact on these processes. These opposing outcomes stem from the distinct infection modes (lytic vs. lysogenic) of the two virus types targeting ANME-2d. The finding offers insight that simultaneous suppression of methane emission and As contamination in rice paddies may be realized through viral application (Wang et al., 2025). Mechanistically, this viral approach targets the methane-dependent As(V) reduction pathway (Section 4.2), showing how manipulating a specific coupling node can yield co-benefits. Furthermore, the application of bacteria that efficiently utilize CH_4_ and promote plant growth can cut CH_4_ emissions. By oxidizing CH₄, these methanotrophic bacteria reduce the electron pool available for downstream As(V) reduction, linking their GHG-mitigating function directly to suppression of As mobilization. Similarly, inoculating arbuscular mycorrhizal fungi reduces N₂O emissions from rice paddies by enhancing plants’ nitrogen uptake (Qian et al., 2023).

Rational fertilization management, involving balanced nitrate-ammonium nitrogen ratios, slow-release fertilizers, deep nitrogen placement, and straw-organic matter amendments coupled with water regulation, can effectively suppress As mobilization and reduce GHG fluxes. Site-specific soil amendments, such as iron or manganese oxides and carbon-based biochar materials, can immobilize soil-bound As and mitigate GHG production (Qi et al., 2025).

Overcoming As pollution requires soil restoration, which refers to the practice of rehabilitating degraded, damaged, or destroyed soil via biological, chemical, and physical methods (Wan et al., 2019). Meanwhile, those strategies exert distinct influences on soil GHG cycling. Most phytoremediation, chemical amendment, and physical remediation techniques currently focus primarily on As immobilization and removal, while their potential impacts on paddy GHG emissions remain poorly understood and require further targeted investigation.

Most phytoremediation, chemical amendment, and physical remediation techniques currently focus primarily on As immobilization and removal, while their potential impacts on paddy GHG emissions remain poorly understood and require further targeted investigation. Among chemical approaches, iron- and manganese-based amendments are particularly relevant to coupling mechanisms: Fe(III) oxides serve as electron acceptors that compete with As(V) and CO₂ for reducing equivalents, while Mn oxides chemically oxidize As(III) to As(V) and also influence N₂O emissions through denitrification pathways (Section 4.3). Biochar amendments present a more complex case—their porous structure can adsorb As(V) and provide habitat for methanotrophs, but labile C fractions may also stimulate methanogenesis, illustrating a potential trade-off that requires context-specific evaluation. Phytoremediation uses natural or genetically engineered plants to remove contaminants. For example, arsenic enters rice from soil through two distinct transporter families. As(V) is taken up via phosphate transporters (PHT1;1) and subsequently reduced to As(III) by As (V) reductase (Ars2). The resulting As(III) can be either taken into the cell or extruded via glycerol transporters (NIP1;1). Once inside, As(III) forms complexes with thiol-rich peptides, which are subsequently transported across the tonoplast into vacuoles by ATP-binding cassette (ABC) transporters for sequestration, representing the major intracellular As detoxification pathway in plant cells (Preetha et al., 2023) (Figure 8). Thus, rice may be a potential candidate for phytoremediation of paddy soils contaminated with mild to moderate As levels (Zhang X. et al., 2024). However, the GHG implications of phytoremediation—particularly root exudate-stimulated methanogenesis versus enhanced rhizosphere oxidation—remain largely unexplored and warrant dedicated study.

Chemical restoration is a commonly adopted remediation method for paddy soil and includes organic and inorganic matter amendments, such as gypsum, lignite, Iron-based modifiers, manganese (Mn), and biochar. Mn-based amendments could convert As(III) species into As(V) and immobilize As in paddy soil. Studies have found that adding MnOx to paddy soils can strengthen denitrification-based As(III) immobilization and decrease N₂O emissions via the anammox route. Meanwhile, nitrate-reducing As(III) oxidation (NRAO) mediated by microorganisms is considered a viable method to lower the bioavailability and toxicity of As in flooded rice paddies. It achieves this by stimulating microbes to oxidize As(III) to As(V), while denitrification occurs concurrently. During microcosm incubation studies, the combination of these two interventions was found to significantly suppress both As(III) mobilization and N₂O emission (Wang et al., 2024). Different types of iron minerals are extensively utilized to remediate soil contaminated by As. Iron oxides can adsorb large quantities of As (especially As(V)). For example, adding poorly crystalline iron oxides can effectively lower the bioavailability of As; similarly, iron hydroxide-modified coal gangue and ferric sulfate-modified coal gangue also notably reduce the concentration of available As in paddy soil. Moreover, FeOOH addition can boost the formation of iron plaque to decrease As uptake through rice roots, but it has potential downsides, such as impeding nutrient absorption or adversely affecting rhizosphere microorganisms via restricted O_2_ diffusion. Conventional biochar is not an effective amendment for As in paddy rice. Low doses reduce grain As via bio-dilution driven by increased biomass, whereas high doses raise soil solution As by boosting iron-reducing bacteria abundance and facilitating reductive dissolution of iron (oxyhydr)oxides (Zhang X. et al., 2024).

Electrokinetic Migration Remediation (EKR) technology involves applying a direct current electric field to either side of contaminated soil. In this electric field, pollutants in the soil move toward the two electrodes with the goal of extracting pollutants from the soil. Though field-based electrokinetic remediation studies for paddy soil have been carried out, the maximum As removal rates achieved are only 54 and 44.4%, respectively, with remediation periods (over 1 month each) also longer compared to laboratory settings (Kim et al., 2014; Jeon et al., 2015). Despite most existing EKR enhancement methods boosting the migration and accumulation of As in soil, they are unable to fully separate and remove contaminants from the soil and damage the physical and chemical properties of the soil (Liao et al., 2022).

Soil washing is categorized into physical separation and chemical extraction methods. The process of this technology entails mixing contaminated soil with a washing agent that dissolves contaminants bound to soil minerals or colloids. Afterward, solid–liquid separation is used to separate the leachate waste liquid, which contains the dissolved pollutants from the soil, ultimately yielding decontaminated soil (Liao et al., 2022). Alkaline DOC solution from wine production waste was utilized in a study, which resulted in an 88% As removal efficiency in paddy soil (Lin et al., 2017). However, ex situ soil washing requires extensive excavation, leading to substantial costs. Additionally, the impairment of the soil’s inherent characteristics from using large quantities of washing agents cannot be overlooked.

Overall, the effectiveness of a co-mitigation strategy is ultimately determined by its impact on the underlying mechanistic couplings—whether it redirects electron flow away from undesirable pathways, shifts the balance of shared substrates, or modifies redox conditions in a direction that benefits both As immobilization and GHG suppression. Strategies that inadvertently intensify electron supply to methanogens while promoting As(V) reduction (e.g., certain labile organic amendments) may create trade-offs, whereas those that enhance oxidative conditions or introduce alternative electron acceptors (e.g., nitrate, Fe/Mn oxides) tend to achieve synergies. This mechanistic lens should guide future screening and optimization of co-mitigation practices.

## 7 Conclusion

This review comprehensively synthesizes the current knowledge on arsenic (As) migration and transformation, the microbial pathways of CO₂, CH₄, and N₂O production and consumption, the coupled interactions between As behaviors and each GHG, the shared environmental drivers that simultaneously modulate both processes, and the performance and trade-offs of existing co-mitigation strategies. We conclude that As and GHG dynamics are mechanistically interwoven through electron competition between As(V)-reducing bacteria and methanogens, shared enzymatic machinery (e.g., methyltransferases), and common redox-sensitive regulators such as pH and organic matter; hence, they cannot be managed in isolation. However, critical mechanistic gaps remain, particularly regarding the thermodynamic and kinetic rules governing electron partitioning under fluctuating redox conditions, the causal pathways linking As methylation/demethylation to CH₄ production/oxidation, and the different effects of specific organic substrates on As mobility versus N₂O emissions. Furthermore, field-scale simultaneous measurements of As species and all three GHGs are severely lacking, which hinders model validation and trade-off assessment under real conditions. Despite these uncertainties, region-specific co-beneficial practices—e.g., alternate wetting-drying irrigation, nitrate-coupled As(III) oxidation, and Fe/Mn-based amendments—show promise, but their efficacy depends strongly on soil properties, climate, and agronomic context. Future research should prioritize long-term field trials, life-cycle assessments, and the integration of multi-omics and predictive modeling to tailor solutions to local conditions, while carefully avoiding unintended increases in N₂O emissions from nitrate-based interventions.
